# A new approach for microstructure imaging

**DOI:** 10.1038/s41598-022-24176-8

**Published:** 2022-11-15

**Authors:** Benoît Plancoulaine, Allan Rasmusson, Christophe Labbé, Richard Levenson, Arvydas Laurinavicius

**Affiliations:** 1 Faculty of Medicine, Vilnius University, Vilnius, Lithuania; 2 ANTICIPE, INSERM, Cancer Center F. Baclesse, University Caen Normandy, Caen, France; 3 National Center of Pathology, Affiliate of Vilnius University Hospital Santaros Clinics, Vilnius, Lithuania; 4 CIMAP, CEA, CNRS, ENSICAEN, University Caen Normandy, Caen, France; 5 Department of Pathology and Laboratory Medicine, UC Davis Health, Sacramento, CA USA

**Keywords:** Cell biology, Microbiology, Mathematics and computing, Optics and photonics

## Abstract

A recurring issue with microstructure studies is specimen lighting. In particular, microscope lighting must be deployed in such a way as to highlight biological elements without enhancing caustic effects and diffraction. We describe here a high frequency technique due to address this lighting issue. First, an extensive study is undertaken concerning asymptotic equations in order to identify the most promising algorithm for 3D microstructure analysis. Ultimately, models based on virtual light rays are discarded in favor of a model that considers the joint computation of phase and irradiance. This paper maintains the essential goal of the study concerning biological microstructures but offers several supplementary notes on computational details which provide perspectives on analyses of the arrangements of numerous objects in biological tissues.

## Introduction

Several computer-aided designs (CAD) are available for application in the domain of photonics and improving their performance depend mainly on the computer power available^1^. The effects of photonics are usually modeled and implemented based on either the Fourier transform or numerical integration algorithms. The fast Fourier transform (FFT) is relatively easy to compute but performance is still limited by high memory demands^2^. The finite difference method in the time-domain^1^ is another efficient algorithm which can be applied to both linear and non-linear optics^3^. In particular, the finite difference method has been used to implement photonic modeling based on Mie’s theory applied to electromagnetism^4^. However, mixed algorithms combining both approaches can be developed to simulate specific effects of photonics. The introduced paper is founded on such a combination of distinct algorithmic approaches^1^.

This paper first gives a review of several algorithms derived from known light-based phenomena accompanied by simulations in order to highlight well-suited computational techniques and address optimal algorithm identification. Subsequently, we describe a newly derived application of Gosse’s algorithm^5^ adapted to biological tissue analysis in MUSE images^5^. Lastly, the supplementary documents for mathematical support are given.

### High frequency techniques for imaging of micro structures

**Figure 1:**
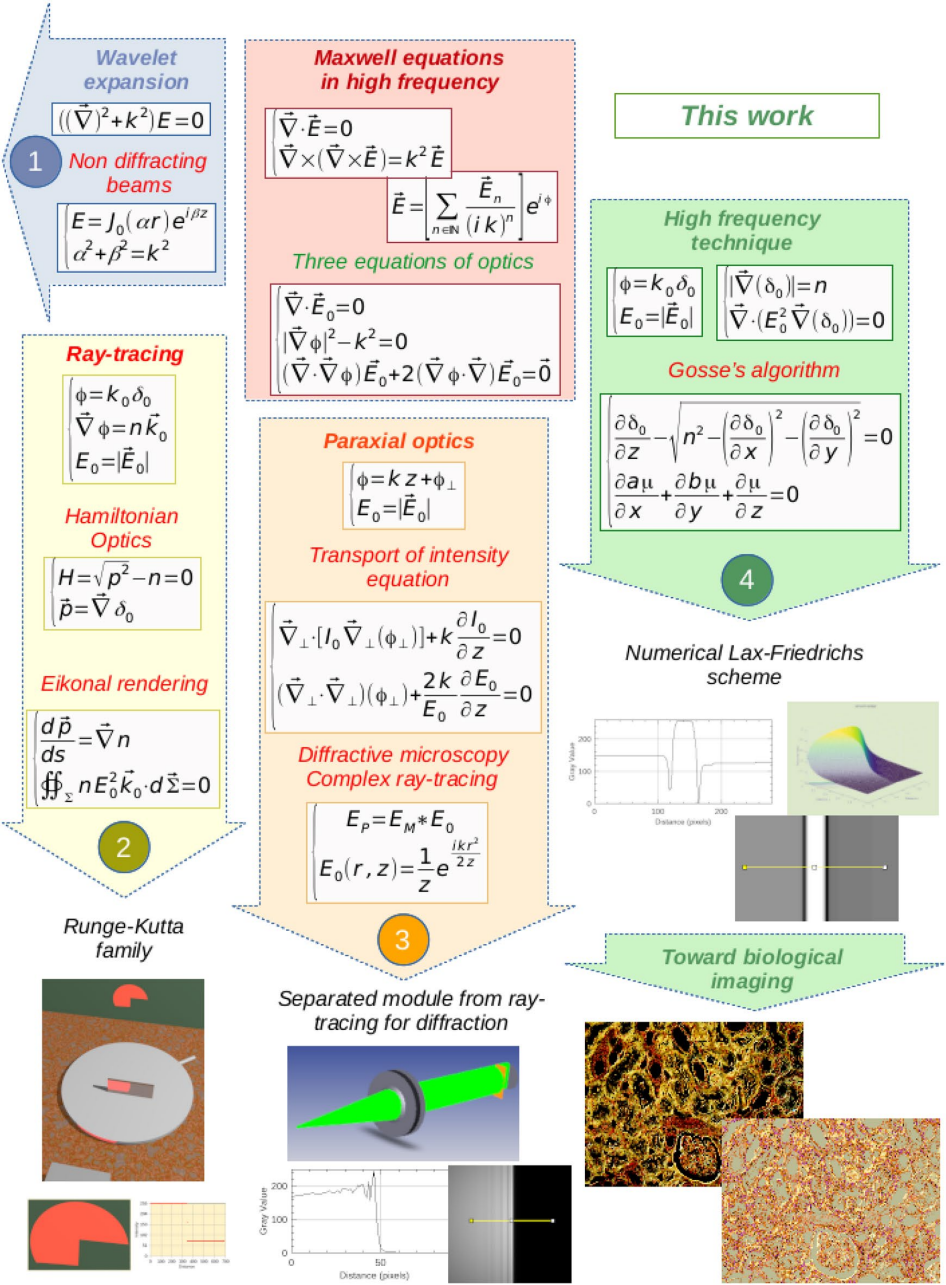
Three computational methods (2, 3, 4) deployed to study photonics-based phenomena.

The relevant electrodynamic phenomena can be described in terms of Maxwell equations^6^. Despite simple formulation, their solutions are complex^7^. However, several computational methods are preferred when frequencies involved include a range in the few hundreds of terahertz, and in this case, asymptotic solutions^6^ can adequately solve the issues posed by optics (Fig. 1).

The field of light-based science called optics can take advantage of asymptotic approximations. To better understand the limits of each approximation, we performed simulations that addressed a simple but revealing mathematical model involving a perfectly conducting half-plane, previously solved analytically by Sommerfeld^8^. In particular, illustrations were produced to highlight the diffraction phenomena. It should be noted that the illustrations are shown enlarged in the supplementary notes (Fig. 1).

The area in blue (Fig. 1(1)) depicts a possible solution showing a wavelet that has an independent envelope in the propagation direction. One of the first solution sets is based on the use of soliton waves, which are solutions to Schrödinger's equation. Solving this last equation is equivalent^5^ to solving the transport and Eikonal equations^9^. It is useful to note that Bessel beams demonstrate the same behavior^7^.

Inside the red area (Fig. 1), the high frequencies included in the illumination help simplify the equations without discarding important vector characteristics^9^. Three equations are thus obtained: the first shows a transverse wave, the second shows the light rays commonly called geometric optics, and the third gives the amplitude of the electric field of the light. These three differential relationships form the basis of optics theory.

The area in yellow (Fig. 1(2)) illustrates application of the two laws of geometric optics^10^ with ray-tracing equations and the conservation principle governing light flux. The Hamilton–Jacobi equation allows for establishing the ray equations through gradient-index (GRIN) media^11^ or establishing an efficient light propagation technique using adaptive wavefront tracing^12^. The numerical computations are carried out by means of a simple Euler forward scheme or an integration method from the Runge–Kutta family^13^.

The area shown in light orange (Fig. 1(3)) relies on determination of the path of light wave propagation, with application of paraxial optics estimations^14^. First, the transport intensity equation^15^ describes the irradiance planes along the optical axis and can be used to analyze the wave fronts and find phase images^14^ (Suppl. Fig. S1). Second, the transport paraxial equation, similar to the well-known Helmholtz’s paraxial wave equation^16^, explains the diffraction of Fresnel’s formula, describes near-plane wave propagation^17^, and computes the Gaussian beam characteristics^14^. A separate module from ray-tracing software^18^ exploits the transport paraxial equation to demonstrate diffraction introduced by optical devices.

The area depicted in green (Fig. 1(4)), represents another elegant approach for studying light propagation associated with optics equations. This area begins with a diffraction study of a perfectly conducting rectangular half-plane^19^ (Suppl. Fig. S3) solved classically by Sommerfeld theory^8^ and showing the importance of determining phase and amplitude of relevant light waves simultaneously. This method can be extended to capture phenomena described by a Hamilton–Jacobi type equation^5,20^. The numerical Lax-Friedrichs scheme is well adapted to address solution convergence^5^.

Based on these different studies, the final algorithm^5,21^, offering the possibility of simultaneously determining the phase and amplitude, is retained in this paper to characterize microstructures of potential interest in a variety of fields.

## Results

### Initial results on simple water bubbles in air

Gosse’s algorithm was programmed in C (GCC) to obtain pyramidal TIFF images (stacks), Octave software^22^ was deployed to generate 2D graphs, along with Povray software^23^ and ImageJ software^24^ with the Volume Viewer plugin^25^ for 3D scenes. A 2D tool was first developed and then extended to 3D space in order to build 3D irradiance illustrations.

This software prototype uses the radius, the 3D coordinates of the center and the refractive index of spherical water bubbles^4,5^, which are entered into a text file. The user can change the default parameters given by the space height (*z_max*) and the sampling step (*z_step*) according to the desired spatial resolution. The process begins at the bottom level (*z* = 0) by initialization according to a monochromatic plane wave (*532 nm by default*) and it propagates the light by computing the phase and the amplitude together at each successive z step up to the top (*z_max*). Therefore, an image stack is constructed that can be analyzed plane by plane or projected with a 3D viewer.

**Figure 2:**
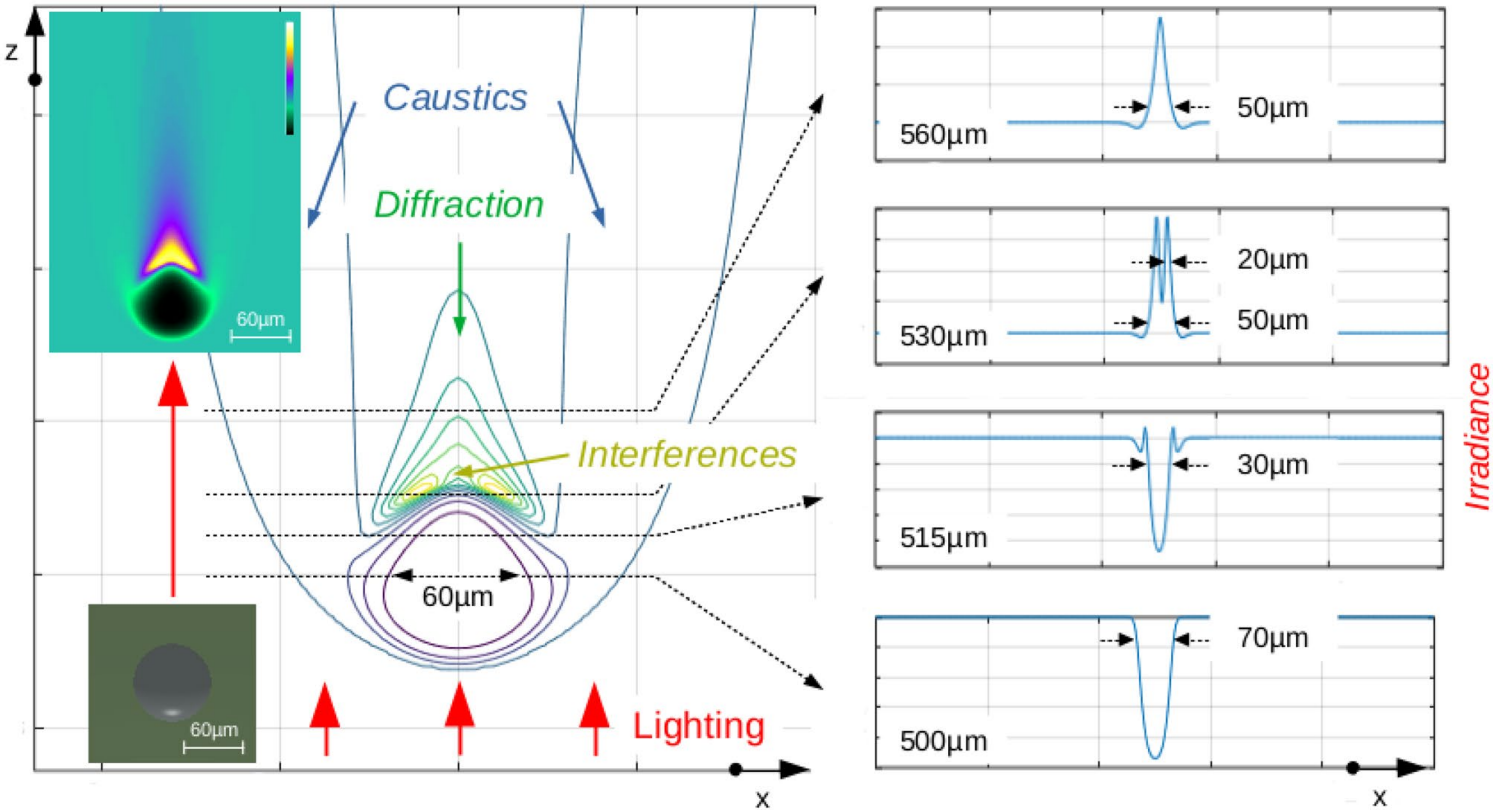
Single water bubble at the left, the irradiance contour in an “xz” plane and at the right, several irradiance profiles (arbitrary scale).

First, one water bubble in air was studied to investigate common light effects (Fig. 2).

A single water bubble was illuminated from the bottom by a parallel-ray beam. Phase and amplitude were computed together in the 3D space (Fig. 2). In the longitudinal plane “xz”, small caustic effects appear at left and right along the bubble surface (in dark blue); the light was focused at the bubble top with an added diffraction effect in green, and two effects of surface interference appear in yellow. The 3D illustration was built in false color by using the "cool" LUT of ImageJ^24^ and the Volume Viewer plugin^25^.

Common optics effects were simulated by means of the numerical Lax–Friedrichs scheme^20^, and were simultaneously computed by means of asymptotic optics equations.

**Figure 3:**
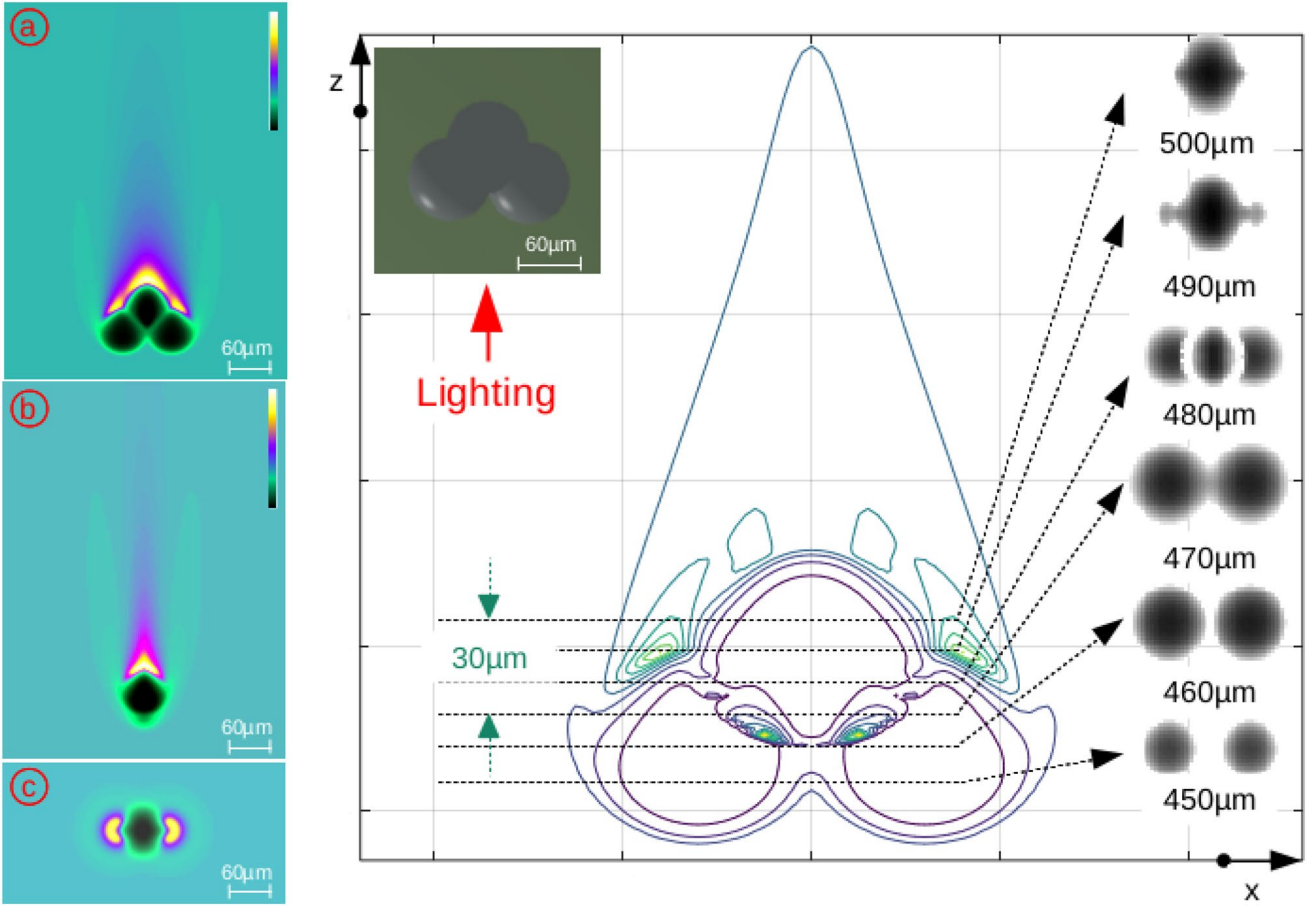
Three water bubbles: at the left, (**a**) “xz” plane, (**b**) “yz” plane and (**c**) “xy” plane, and at the right, the irradiance contour in an “xz” plane with several images in gray levels (*arbitrary Y scale*).

Three water bubbles adjacent to one another were then considered, illuminated using a parallel-ray beam in order to check the extension of this approach in 3D (Fig. 3).

The 3D version of this tool^20^ computed the phase and amplitude together in volume and extracted transverse planes. Each transverse plane containing irradiance was then translated to a gray-level 8-bit encoded image.

### Biological tissue modeled as bubble complex distributions

Biological tissue analyses are based on irradiance images^26^ using suitable staining to highlight particular tissue structures of interest. However, substantial physical information is contained in the phase of the light wave^27^ and analyses of irradiance is incomplete^15^ as typically tissue staining behavior is captured using only absorbance-based measurements. Therefore, there is potential utility in computing images that highlight the amplitude related to the phase^28^. This enhanced approach could improve information content while requiring fewer stain-based procedures. This reconstruction is just what is made possible by the use of Gosse's algorithm^5^ which overcomes some limitations of paraxial optics such as image reconstruction based on the intensity transport intensity equation^10,15^.

**Figure 4:**
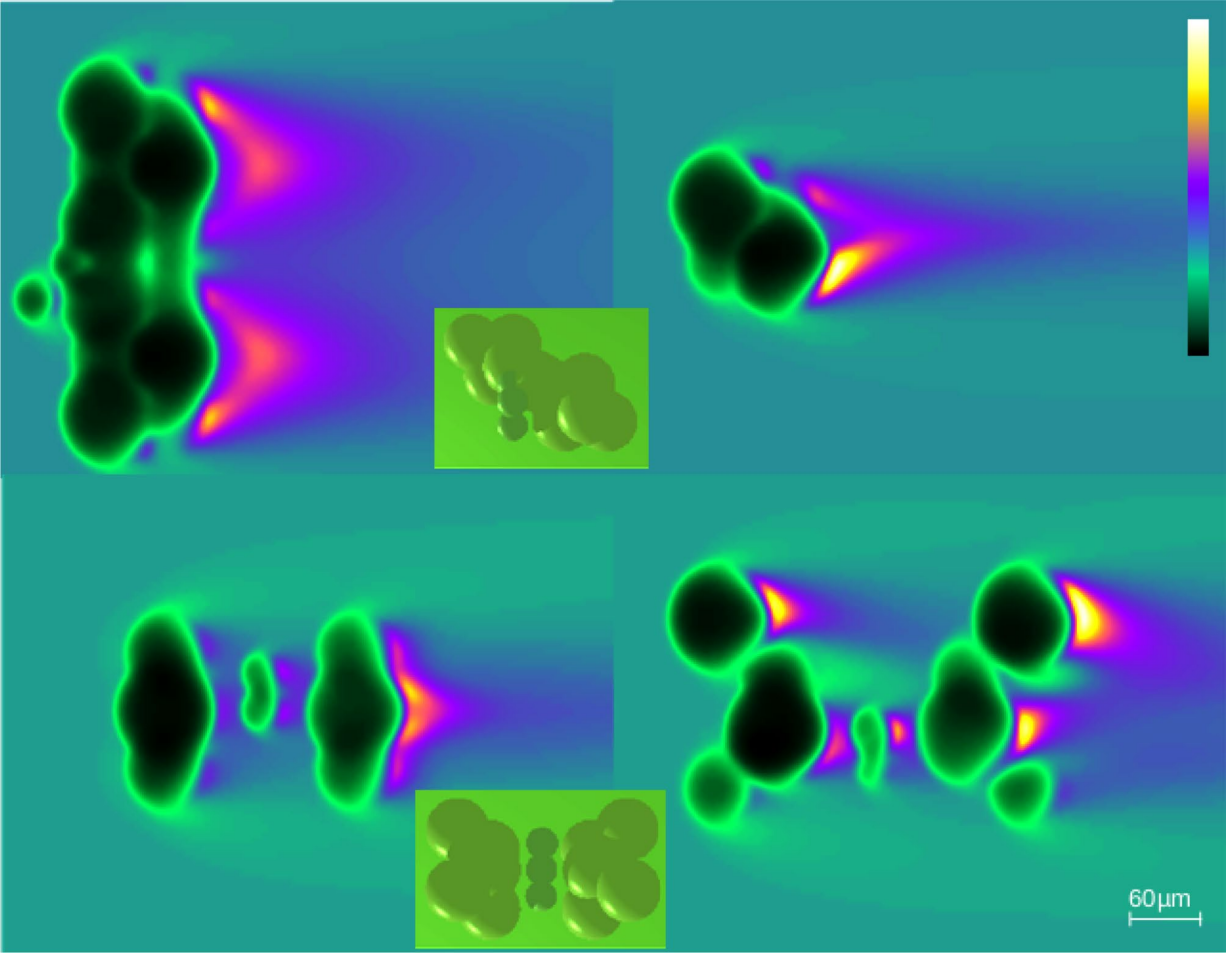
Two complex water bubble distributions (left: “xz” planes, right: “yz” planes).

Biological tissues appear as heterogeneous complex structures when studied at the scale of tens of micrometers, considerably larger than the wavelength of visible light waves, which are a few hundreds of nanometers in wavelength. It is important to consider that when tissue is illuminated by a beam of parallel rays, many reflections and refractions can occur in even within a thin biological specimen. These produce stigmatic rays, which can create microelement images, as well as astigmatic rays, which create caustics^29^. In particular, the nuclei inside cells, and the cells themselves, can act as microlenses or microbubbles^30^ (Fig. 4).

Therefore, improving microscope lighting could enhance tissue analysis for biologists and pathologists. Light effects can generate different appearances in the same object and can impact computer vision by generating unanticipated or complex data.

**Figure 5:**
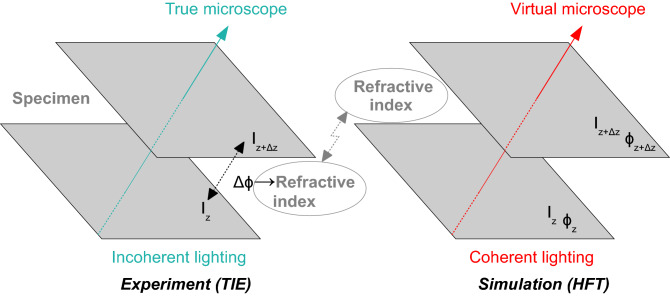
Left: experiment with a microscope using incoherent lighting. Right: image simulation using coherent lighting.

The application of Gosse's algorithm^5^ allows for computing new illuminations of the tissues. However, these operations require estimation of the refraction index from several intensity planes *I*_*z*_ (Fig. 5).

The refractive index is computed from the change of the phase *ϕ* given by the the classical transport intensity equation^14,15^ (TIE). Therefore, this estimation allows propagation of the light in the tissues by means of the high frequency technique (HFT).

### Application in microscopy with ultraviolet surface excitation

**Figure 6:**
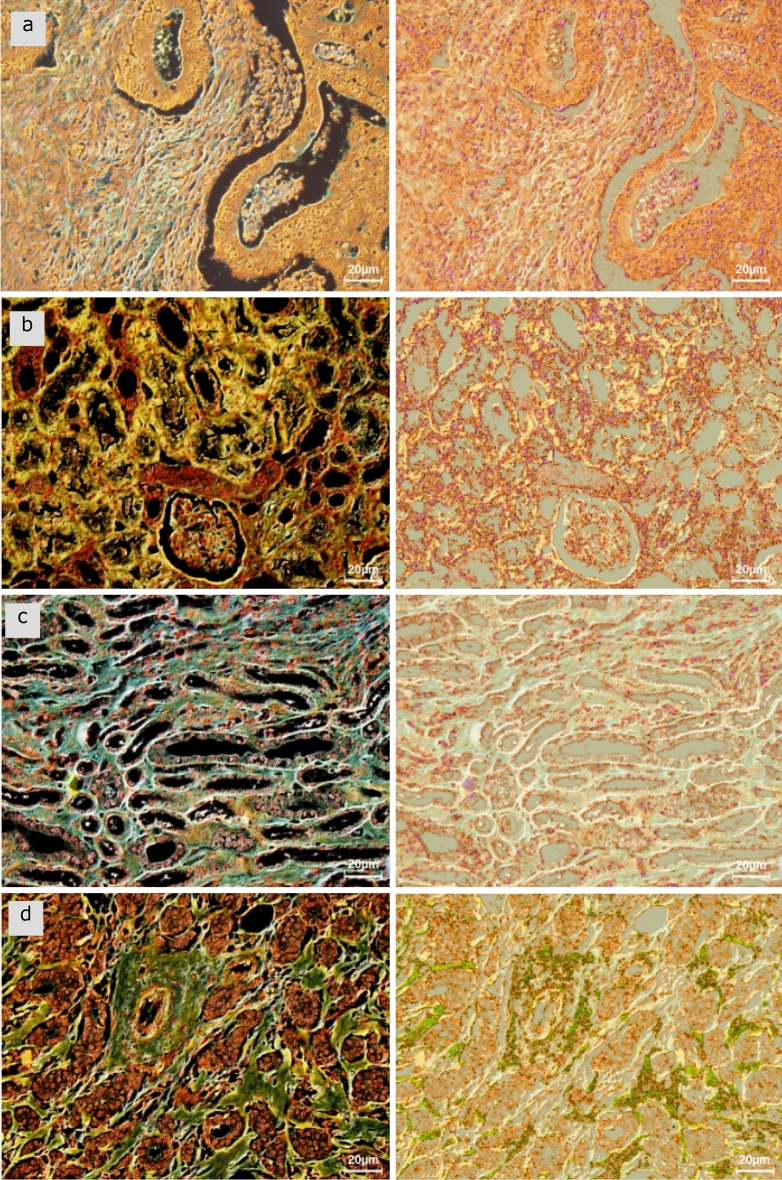
At the left, four digital images acquired by means of a MUSE microscope of Hoechst and rhodamine-stained 5-µm sections, and at the right, same images superimposed with the images processed by the high frequency technique: (**a**) slice of colorectal cancer tissue, (**b**) slice of renal medulla (normal), (**c**) slice of renal cortex (normal), (**d**) slice of breast cancer tissue.

In Microscopy with Ultraviolet Surface Excitation (MUSE) technique, UV excitation deploys wavelengths whose central wavelengths range between 275 and 285 nm in order to acquire highly resolved tissue images captured using either sectioned (on slides—as shown here) or unsectioned (slide-free) tissue specimens^31^. However, the interactions between fluorescence lighting and biological tissue are also complex processes. Indeed, some randomly distributed tissue components can remit the light in all directions, a subset of which are captured by the microscope objective. These multiple light sources are amplified and create caustic effects. Consequently, the light waves in the sections are scrambled by the caustic effects, and analysis becomes more difficult (Fig. 6).

First, the refractive index is estimated from the remitted broad-spectrum light by the specimen in the visible spectrum. Then the high frequency technique simulates new lighting of the biological specimen with monochromatic plane waves taking the refracting index into account. These new images superimposed onto the corresponding MUSE images highlights some otherwise inconspicuous details that arise due to caustic and diffraction effects.

## Conclusion and discussion

Many paths^32^ in the world of optics are possible to describe computational methods with^33^ or without^5^ the use of light ray models. Different algorithms^34^ can assist depending on the optical equations that are used (Fig. 1): approach 2 (Fig. 1(2)) exploits the two laws of geometric optics that are limited to diffraction and interference analysis; approach 3 (Fig. 1(3)) exploits light wave propagation laws and is limited mainly by the combination of plane waves; and approach 4 (Fig. 1(4)) exploits optical equations to build phase and amplitude images without dependence on modeling of virtual light rays.

Approach 4 appears to be the most promising because it directly accounts for the properties of light waves by considering computation performed with asymptotic optics equations and reveals caustic and diffraction phenomena. Moreover, its numerical solution is equivalent to Schrödinger’s equation, which can describe nondiffracting waves. This last method addresses both irradiance and phase images, which are necessary for the full characterization of biological tissue slices^21^.

A simple model of water bubbles allowed us to check whether the task could be solved directly, taking into account their relative arrangements in 3D space. This model, based on Gosse’s algorithm^5^, is now translated to process digital image stacks in order to understand lighting in biological tissue slices.

This high frequency technique adapted to biological tissues will be evolved for multiple lighting situations (several wavelengths, other spatial distributions) and tested with multiple staining approaches. These experiments will use classical microscopes as well as recently developed whole-slide scanners (used to create large gigapixel images) now being deployed in clinical histopathology applications.

## Supplementary Information

Supplementary Information 1.

Supplementary Information 2.

Supplementary Information 3.

Supplementary Information 4.

Supplementary Information 5.

## Data Availability

The datasets used and/or analyzed during the current study available from the corresponding author on reasonable request.
