# Supplementary material for: A new approach for microstructure imaging
Source: Sci Rep. 2022 Nov 15;12:19565. doi: 10.1038/s41598-022-24176-8 (PMC9666525; doi:10.1038/s41598-022-24176-8)
Supplement: Supplementary file 1 — Supplementary Information 1. [file 41598_2022_24176_MOESM1_ESM.pdf]

Three equations of the optics

Benoît Plancoulaine[1,2,*], Allan Rasmusson[1,3], Christophe Labbé[4], Richard Levenson[5], Arvydas Laurinavicius[1,3].

1 Institute of Biomedical Sciences, Faculty of Medicine, Vilnius University, Vilnius, Lithuania.

2 ANTICIPE, INSERM, University Caen Normandy, Cancer Center F. Baclesse, Caen, France.

3 National Center of Pathology, Affiliate of Vilnius University Hospital Santaros Clinics, Vilnius, Lithuania.

4 CIMAP, CEA, CNRS, ENSICAEN, University Caen Normandy, Caen, France.

5 Department of Pathology and Laboratory Medicine, UC Davis Health, Sacramento, CA, USA.

Introduction

Optics equations attributable to intrinsic space-time properties (Minkowski space) are defined by a potential vector whose changes induce an electromagnetic field, which is noted by $\vec{E}$ for the electric field and $\vec{B}$ for the magnetic field. However, optical measurements are taken by means of sensors sensitive to the irradiance, which is expressed as the squared norm of the electric field. Thus, optical phenomena are explained by equations containing only the electric field $\vec{E}$.

Lüneburg-Kline's series expansion

The time harmonic approach is used to find optics solutions from Maxwell equations[1]. Therefore, electric field $\vec{E}$ analyses by frequency can be expressed in Fourier space[2] by the differential system (1).

$$\begin{cases} \vec{\nabla}\cdot\vec{E}=0 \\ \vec{\nabla}\times(\vec{\nabla}\times\vec{E})=k^2\vec{E} \end{cases} \quad (1)$$

where $\vec{\nabla}$ is the "*nabla operator*" or *the gradient* and $k=\frac{2\pi}{\lambda}$ is the modulus of the wave vector depending of the wavelength λ.

Exact solutions to Maxwell equations are generally very difficult to obtain, so approximate solutions to the boundary conditions by asymptotic expansion are used in practice[3]. One strategy, which is well suited for optical phenomena occurring for high-frequency waves (*hundreds of Tera Hertz*), is approximation by Lüneburg-Kline series expansion[3] given by (2).

$$\vec{E}(r,\omega)=\vec{E}_{\Sigma}\,e^{i\phi}=\sum_{p\in\mathbb{N}}\frac{\vec{E}_p(r)}{(i\omega)^p}\,e^{i\phi(r)} \text{ for } \omega\rightarrow\infty \quad (2)$$

where r is the coordinate of a point in physical space, $\vec{E}_p(r)$ is the electric field of the p' order, i is the complex "i", and ϕ is the linear phase according to ω.

Fundamental mode in optics

Although the aim in optics is to approximate the first term $\vec{E}_0\, e^{i\phi}$ of the Lüneburg-Kline series expansion[3], called "the fundamental mode", the expansion is shortened to two terms $\vec{E}=\vec{E}_\Sigma\, e^{i\phi}=\left(\vec{E}_0-\frac{i}{\omega}\,\vec{E}_1\right) e^{i\phi}$ for calculation purposes. This reduced expansion is included in the first differential equation of (1) to give a new differential system (3).

$$\begin{cases}\vec{\nabla}\phi\cdot\vec{E}_0=0\\ \vec{\nabla}\cdot\vec{E}_0+\frac{1}{\omega}\vec{\nabla}\phi\cdot\vec{E}_1=0\end{cases}\quad(3)$$

where $\vec{\nabla}\phi$ is the gradient operator of the phase ϕ, which is also of power one since ϕ depends linearly on ω. The first equation of (3) expresses the transversal propagation of the field $\vec{E}_0$, and the second equation expresses the relationship between the two fields $\vec{E}_0$ and $\vec{E}_1$, which are used hereafter to derive the other optics equations.

Phase expressed by the eikonal equation

Substituting the reduced expansion of the electric field $\vec{E}=\vec{E}_\Sigma\, e^{i\cdot\phi}$ (2) into the second equation of (1) gives (4) with $\vec{\nabla}\times\vec{E}=\vec{\nabla}\times\vec{E}_\Sigma e^{i\cdot\phi}+i\vec{\nabla}\phi\times\vec{E}_\Sigma\, e^{i\cdot\phi}$.

$$\vec{\nabla}\times(\vec{\nabla}\times\vec{E}_\Sigma)+i\vec{\nabla}\phi\times(\vec{\nabla}\times\vec{E}_\Sigma)+i\vec{\nabla}\times(\vec{\nabla}\phi\times\vec{E}_\Sigma)-\vec{\nabla}\phi\times(\vec{\nabla}\phi\times\vec{E}_\Sigma)-k^2\vec{E}_\Sigma=\vec{0}\quad(4)$$

The term $\vec{\nabla}\times(\vec{\nabla}\times\vec{E}_\Sigma)$ includes ω of zeroth power and can be eliminated. The term with ω of power one is (5).

$$\vec{\nabla}\phi\times(\vec{\nabla}\times\vec{E}_0)+\vec{\nabla}\times(\vec{\nabla}\phi\times\vec{E}_0)+\vec{\nabla}\phi\times\left(\vec{\nabla}\phi\times\frac{\vec{E}_1}{\omega}\right)+k^2\frac{\vec{E}_1}{\omega}=\vec{0}\quad(5)$$

and the term $\vec{\nabla}\phi\times(\vec{\nabla}\phi\times\vec{E}_0)+k^2\vec{E}_0=\vec{0}$ which contains ω of second power can be simplified using the first equation of (3) to give the first major result (6).

$$|\vec{\nabla}\phi|^2-k^2=0\quad(6)$$

This equation (6) named the "eikonal equation" governs geometric optics.

Fundamental mode expressed by the transport equation

Using the two identities $\vec{\nabla}\phi\cdot\vec{\nabla}\phi-k^2=0$ (6) and $\vec{\nabla}\cdot\vec{E}_0+\frac{1}{\omega}\vec{\nabla}\phi\cdot\vec{E}_1=0$ (3), the previous term (5) can be expressed as (7).

$$\vec{\nabla}\phi\times(\vec{\nabla}\times\vec{E}_0)+\vec{\nabla}\times(\vec{\nabla}\phi\times\vec{E}_0)-(\vec{\nabla}\cdot\vec{E}_0)\cdot\vec{\nabla}\phi=\vec{0}\quad(7)$$

The successive use of two classical vectorial identities (8)

$$\begin{cases}\vec{\nabla}\times(\vec{x}\times\vec{y})=(\vec{\nabla}\cdot\vec{y})\vec{x}-(\vec{\nabla}\cdot\vec{x})\vec{y}+(\vec{y}\cdot\vec{\nabla})\vec{x}-(\vec{x}\cdot\vec{\nabla})\vec{y}\\ \vec{\nabla}(\vec{x}\cdot\vec{y})=\vec{x}\times(\vec{\nabla}\times\vec{y})+\vec{y}\times(\vec{\nabla}\times\vec{x})+(\vec{x}\cdot\vec{\nabla})\vec{y}+(\vec{y}\cdot\vec{\nabla})\vec{x}\end{cases}\quad(8)$$

with $\vec{x}=\vec{\nabla}\phi$ and $\vec{y}=\vec{E}_0$, reduces term (7) into (9).

$$\vec{\nabla}\phi\times(\vec{\nabla}\times\vec{E}_0)-(\vec{\nabla}\cdot\vec{\nabla}\phi)\vec{E}_0+(\vec{E}_0\cdot\vec{\nabla})\vec{\nabla}\phi-(\vec{\nabla}\phi\cdot\vec{\nabla})\vec{E}_0=\vec{0} \quad (9)$$

Using the first equation of (3), the second major result (10) is obtained.

$$(\vec{\nabla}\cdot\vec{\nabla}\phi)\vec{E}_0+2(\vec{\nabla}\phi\cdot\vec{\nabla})\vec{E}_0=\vec{0} \quad (10)$$

The “transport equation” (10) allows for computing the fundamental mode $\vec{E}_0$ of the electric field.

References

1. Born (M.), Wolf (E.), Principles of Optics, Book, 6th edition, *Pergamon Press , Inc* (1980).

2. Goodman (J.W.), Introduction to Fourier Optics, Editions McGraw-Hill, New York, Book 2st edition (1996).

3. Kline (M.), Electromagnetic theory and Geometrical optics, Institute of mathematical sciences, New York University, Research report EM-171 (1962).
