# Supplementary material for: A new approach for microstructure imaging
Source: Sci Rep. 2022 Nov 15;12:19565. doi: 10.1038/s41598-022-24176-8 (PMC9666525; doi:10.1038/s41598-022-24176-8)
Supplement: Supplementary file 2 — Supplementary Information 2. [file 41598_2022_24176_MOESM2_ESM.pdf]

Laws of the geometric optics

Benoît Plancoulaine[1,2,*], Allan Rasmusson[1,3], Christophe Labbé[4], Richard Levenson[5], Arvydas Laurinavicius[1,3].

1 Institute of Biomedical Sciences, Faculty of Medicine, Vilnius University, Vilnius, Lithuania.

2 ANTICIPE, INSERM, University Caen Normandy, Cancer Center F. Baclesse, Caen, France.

3 National Center of Pathology, Affiliate of Vilnius University Hospital Santaros Clinics, Vilnius, Lithuania.

4 CIMAP, CEA, CNRS, ENSICAEN, University Caen Normandy, Caen, France.

5 Department of Pathology and Laboratory Medicine, UC Davis Health, Sacramento, CA, USA.

Introduction

Several ray tracing software programs exploit the two laws of geometric optics considering the eikonal and transport equations. The eikonal equation, being a nonlinear partial differential equation, is integrated using the mathematical method of "characteristics" to find the light ray equations. Then, the transport equation is also integrated using the Green-Ostrogradsky theorem to find the inner irradiance of different objects.

Eikonal and transport equation

The eikonal and transport equation (1) are established in *supplementary note 1*.

$$\begin{cases} |\vec{\nabla}\phi|^2 - k^2 = 0 \\ (\vec{\nabla}\cdot\vec{\nabla}\phi)\vec{E}_0 + 2(\vec{\nabla}\phi\cdot\vec{\nabla})\vec{E}_0 = \vec{0} \end{cases} \quad (1)$$

where $\vec{\nabla}$ is the “nabla operator” or the gradient, $\vec{E}_0$ is the amplitude of the electric filed and ϕ is the wave phase. The wave vector $\vec{k}=\vec{\nabla}\phi$ drives the light rays, and its modulus equals $\|\vec{k}\|=nk_0$ where n is the reflective index and $k_0=\frac{2\pi}{\lambda}$ depends on the wavelength λ.

Descartes’ law of the geometric optics

These ray tracing software programs exploit the nonlinear eikonal equation in order to find the parametric equations of light rays. To obtain variables independent of the dielectric medium where the light propagates, the optical path $\delta_0=n\delta$ is defined as the equivalent path followed by light in a vacuum. The optical path depends on the real path δ followed by light and the refractive index $n=\frac{c_0}{c}$ of the dielectric medium, where c_0 is the speed of light in a vacuum. The phase is rewritten according to $\phi=k_0\delta_0$ and the eikonal

equation according to $(\vec{\nabla}\delta_0)^2=n^2$ with $k=nk_0$. To search the optical path δ_0, the eikonal equation is written as a Hamilton-Jacobi equation[1] $H=\sqrt{p^2}-n=0$, where H is the Hamiltonian and $\sqrt{p^2}=\|\vec{p}\|$ is the norm of the vector $\vec{p}=\vec{\nabla}\delta_0$. The solution δ_0 depending on the parameter s is computed from the first-order partial differential equation system (2), which is adapted by introducing the partial differential operations of the Hamiltonian H.

$$\begin{cases} \frac{dx}{ds}=\frac{\partial H}{\partial p_x}, \frac{dy}{ds}=\frac{\partial H}{\partial p_y}, \frac{dz}{ds}=\frac{\partial H}{\partial p_z} \\ \frac{dp_x}{ds}=-\frac{\partial H}{\partial x}, \frac{dp_y}{ds}=-\frac{\partial H}{\partial y}, \frac{dp_z}{ds}=-\frac{\partial H}{\partial z} \\ \frac{d\delta_0}{ds}=p_x\frac{\partial H}{\partial p_x}+p_y\frac{\partial H}{\partial p_y}+p_z\frac{\partial H}{\partial p_z} \end{cases}, \begin{cases} \frac{dx}{ds}=\frac{p_x}{p}, \frac{dy}{ds}=\frac{p_y}{p}, \frac{dz}{ds}=\frac{p_z}{p} \\ \frac{dp_x}{ds}=\frac{\partial n}{\partial x}, \frac{dp_y}{ds}=\frac{\partial n}{\partial y}, \frac{dp_z}{ds}=\frac{\partial n}{\partial z} \\ \frac{d\delta_0}{ds}=p \end{cases} \quad (2)$$

Similarly, system (3) is obtained by mixing the two first lines to achieve the common Descartes' law[1] described to the second line.

$$\begin{cases} p_x=n\frac{dx}{ds}, p_y=n\frac{dy}{ds}, p_z=n\frac{dz}{ds} \\ \frac{d}{ds}\left(n\frac{dx}{ds}\right)=\frac{\partial n}{\partial x}, \frac{d}{ds}\left(n\frac{dy}{ds}\right)=\frac{\partial n}{\partial y}, \frac{d}{ds}\left(n\frac{dz}{ds}\right)=\frac{\partial n}{\partial z} \\ \frac{d\delta_0}{ds}=n \end{cases} \quad (3)$$

Therefore, the eikonal equation allows for explaining the light path and the light ray orientation. This algorithm, from a Hamilton-Jacobi equation, is the computing base of ray tracing software.

Irradiance law of the geometric optics

These ray tracing software programs use the irradiance law of geometric optics to compute the object lighting considering the integration of the transport equation (1). When the components of the electric field are independent, the scalar amplitude E_0 is introduced in Equation (1), which leads to a new simplified equation (4) after multiplication by E_0.

$$\vec{\nabla}\cdot(E_0^2\vec{\nabla}\phi)=0 \quad (4)$$

where $I_0=E_0^2$ is the irradiance.

Herein, the volume used to apply the Green-Ostrogradsky theorem is a tube drawn by light rays[2], which is closed at its ends by two wave surfaces Fig. S1).

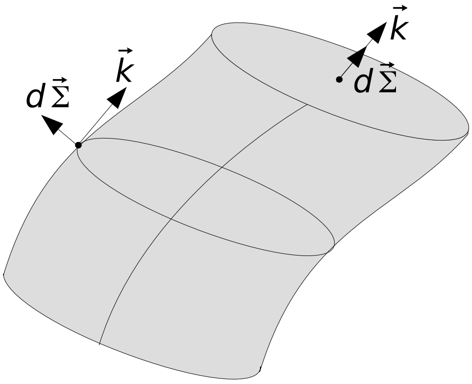

Fig. S1: Tube drawn by light rays.

The integral is obtained by $\oiint_{\Sigma} E_0^2 \vec{\nabla}\phi \cdot d\vec{\Sigma} = 0$ applying this theorem, where Σ is the closed surface and $d\vec{\Sigma}$ is an infinitesimal surface oriented outwards. This integral is split into three parts: the first part, computed on the body of the tube, is null because the surface external vector is orthogonal to the wave vector $\vec{k}$; the second part, expressed by $\iint_{\Sigma_1} E_0^2 n k_0 d\Sigma$, has the surface external vector aligned to the wave vector, and the third part, expressed by $\iint_{\Sigma_2} E_0^2 n k_0 d\Sigma$, has an opposite sign to the previous one because the orientation of the surface external vector is inverted. Therefore, the equality $\iint_{\Sigma_2} E_0^2 n d\Sigma = \iint_{\Sigma_1} E_0^2 n d\Sigma$ is carried out, where Σ_1 and Σ_2 are the first and second wave surfaces. The integration result leads to Equation (5) when the quantities are uniform on the wave surfaces.

$$E_0(\Sigma_1) = E_0(\Sigma_2)\sqrt{\frac{n_1 \Sigma_1}{n_2 \Sigma_2}} \quad (5)$$

where n_1 and n_2 are the reflexive indexes on the first and second wave surfaces. The optical index is constant in a homogeneous medium $n_2 = n_1$, and therefore, the equation transport shows a preserved light flux[2].

Ray tracing for a perfectly conducting rectangular half plane

These ray tracing methods, driven by the two laws of the geometric optics, are able to build an image[3] that outlines the shape of a perfectly conducting rectangular half-plane (Fig. S2).

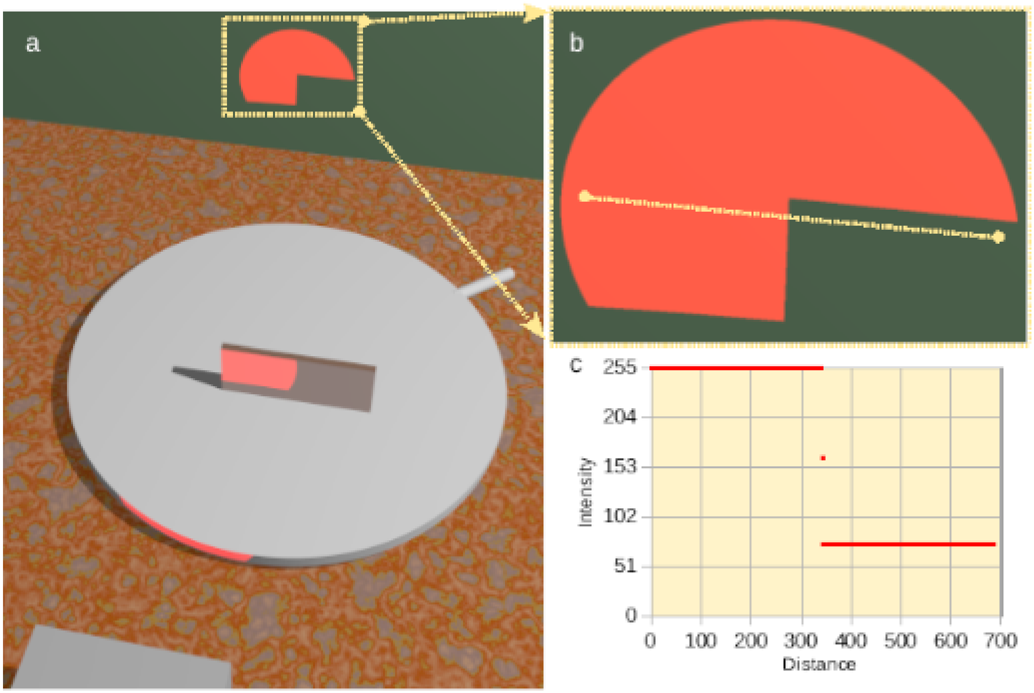

Fig. S2: Amplitude of the perfectly conducting half plane[3].

Unfortunately, the high image resolution does not allow for distinguishing diffraction effects (Fig. S2).

References

1. Fatemi (E.), Engquist (B.), Osher (S.), Numerical Solution of the High Frequency Asymptotic Expansion of the Scalar Wave Equation, Journal of Computational Physics, 120:145-155 (1995).

2. Balanis (CA.), Advanced engineering electromagnetics, Book, 2nd edition, John Wiley & Sons, Inc (2011).

3. Povray Software, Persistence of Vision (TM) Raytracer, Ltd., Williamstown, Victoria, Australia (2004).
