# Supplementary material for: A new approach for microstructure imaging
Source: Sci Rep. 2022 Nov 15;12:19565. doi: 10.1038/s41598-022-24176-8 (PMC9666525; doi:10.1038/s41598-022-24176-8)
Supplement: Supplementary file 3 — Supplementary Information 3. [file 41598_2022_24176_MOESM3_ESM.pdf]

Transport propagation equations

Benoît Plancoulaine[1,2,*], Allan Rasmusson[1,3], Christophe Labbé[4], Richard Levenson[5], Arvydas Laurinavicius[1,3].

1 Institute of Biomedical Sciences, Faculty of Medicine, Vilnius University, Vilnius, Lithuania.

2 ANTICIPE, INSERM, University Caen Normandy, Cancer Center F. Baclesse, Caen, France.

3 National Center of Pathology, Affiliate of Vilnius University Hospital Santaros Clinics, Vilnius, Lithuania.

4 CIMAP, CEA, CNRS, ENSICAEN, University Caen Normandy, Caen, France.

5 Department of Pathology and Laboratory Medicine, UC Davis Health, Sacramento, CA, USA.

Introduction

Several ray tracing software programs exploit propagation equations to simulate the diffraction phenomena, adding a supplementary module or summing astigmatic Gaussian rays. These equations come from the two eikonal and transport equations by applying the assumptions of the paraxial optics.

Paraxial optics

Paraxial optics assumes experiments that only exploit light rays close to the axis and "*almost plane*" wave surfaces. Therefore, the wave propagates mainly as a plane wave along the z axis, but a part of its phase ϕ is corrected by $\phi_\perp$ (1) depending on the coordinates of a transverse plane to the z axis.

$$\phi = kz + \phi_\perp \quad (1)$$

This phase (1) must check the eikonal equation[1] $(\vec{\nabla}\phi)^2 - k^2 = 0$ *(supplementary note 1*), which is computed according to $\vec{\nabla}\phi = k\vec{u}_z + \vec{\nabla}_\perp \phi_\perp$, where $\vec{u}_z$ is the unitary vector of the z axis and $\vec{\nabla}_\perp$ is the reduced operator of the vector differential operator $\vec{\nabla}$ to the coordinates of a transverse plane at the z axis. Thus, the phase is found from $(\vec{\nabla}\phi)^2 = k^2 + (\vec{\nabla}_\perp \phi_\perp)^2$, and the eikonal equation is therefore checked when the part $(\vec{\nabla}_\perp \phi_\perp)^2$ is insignificant by applying the "*almost plane*" assumption.

Paraxial optics is the main assumption used to find the transport intensity equation and the transport paraxial equation.

Transport intensity equation

The transport intensity equation[2] derives from the transport equation (*supplementary note 1*) in paraxial optics. In particular, the transport intensity studies the light flux to compute phase images. For instance, two or three irradiance images of transparent brain cells, with one placed in focus, allows for seeing their varied structures (Fig. S1).

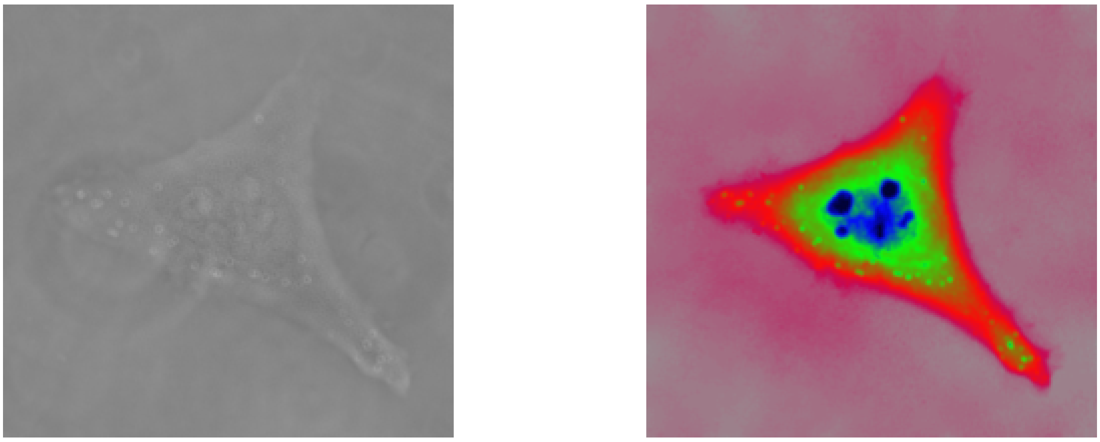

Fig. S1: Phase image of a brain cell[2].

The study of a transport equation, given by $I_0(\vec{\nabla}\cdot\vec{\nabla}\phi)+\vec{\nabla}\phi\cdot\vec{\nabla}I_0=0$ (*supplementary note 2*), starts by means of its expression (2) expanded from the phase $\phi=kz+\phi_\perp$, which is explained in paraxial optics.

$$I_0(\vec{\nabla}_\perp\cdot\vec{\nabla}_\perp\phi_\perp)+\vec{\nabla}_\perp\phi_\perp\cdot\vec{\nabla}_\perp I_0+I_0\left[\frac{\partial^2\phi}{\partial z^2}\right]+\frac{\partial\phi}{\partial z}\frac{\partial I_0}{\partial z}=0 \quad (2)$$

This expression is modified to (3) by replacing the two partial differential parts $\frac{\partial\phi}{\partial z}=k$ and $\frac{\partial^2\phi}{\partial z^2}=0$.

$$\vec{\nabla}_\perp\cdot[I_0\vec{\nabla}_\perp\phi_\perp]+k\frac{\partial I_0}{\partial z}=0 \quad (3)$$

Therefore, the phase image is computed by considering two or three irradiance images, where one is focused for different coordinates z by applying expression (3).

Transport paraxial equation

The transport paraxial equation also derives from the transport equation in paraxial optics. The transport equation is rewritten by the new equation (4).

$$E_0(\vec{\nabla}_\perp\cdot\vec{\nabla}_\perp\phi_\perp)+2\vec{\nabla}_\perp\phi_\perp\cdot\vec{\nabla}_\perp E_0+2k\frac{\partial E_0}{\partial z}=0 \quad (4)$$

using the expressions $(\vec{\nabla}\cdot\vec{\nabla}\phi)=(\vec{\nabla}_\perp\cdot\vec{\nabla}_\perp\phi_\perp)$ and $\vec{\nabla}\phi\cdot\vec{\nabla}E_0=\vec{\nabla}_\perp\phi_\perp\cdot\vec{\nabla}_\perp E_0+k\frac{\partial E_0}{\partial z}$.

Equation (4) can be directly checked with any electrical fields in paraxial optics. However, more commonly, the scalar product $\vec{\nabla}_\perp\phi_\perp\cdot\vec{\nabla}_\perp E_0$ is assumed to be insignificant when the Cartesian coordinates $x\ll z$ with $\left|\frac{\partial E_0}{\partial x}\right|$ and $y\ll z$ with $\left|\frac{\partial E_0}{\partial y}\right|$ are limited.

The transport equation becomes another equation (5) called a transport paraxial equation, which divides into a first part concerning the phase $\phi_\perp$ and a second part concerning the amplitude E_0.

$$(\vec{\nabla}_\perp \cdot \vec{\nabla}_\perp \phi_\perp) + \frac{2k}{E_0}\frac{\partial E_0}{\partial z} = 0 \quad (5)$$

In particular, the Fresnel diffraction formula[1] is the solution for the transport paraxial equation (5).

Moreover, for axially symmetric beams, this last equation is expressed in cylindrical coordinates (6).

$$\frac{1}{r}\frac{\partial}{\partial r}\left(r\frac{\partial \phi_\perp}{\partial r}\right) + \frac{2k}{E_0}\frac{\partial E_0}{\partial z} = 0 \quad (6)$$

This equation can be solved by finding the phase in the form of an expansion (7) according to the even powers of r^{2p}.

$$\phi_\perp = -\frac{kr^2}{2}\sum_{p\in\mathbb{N}}(-1)^p \frac{r^{2p}}{(p+1)^2} f_{2p}(z) \quad (7)$$

where the functions $f_{2p}(z)$ remain to be determined. The integration of the amplitude E_0 with respect to the coordinate z results in an exponential composed of the feature polynomial (8).

$$E_0(r,z) = E_0(r,0)e^{P(r)} \text{ with } P(r) = \sum_{p\in N}(-1)^p r^{2p} \int f_{2p}(z)\,dz \quad (8)$$

where the profile $E_0(r,0)$ of the electric field amplitude is defined in the transverse plane cutting the optical axis in $z=0$. The feature polynomial $P(r)$ drives the type of beam (Fig. S2).

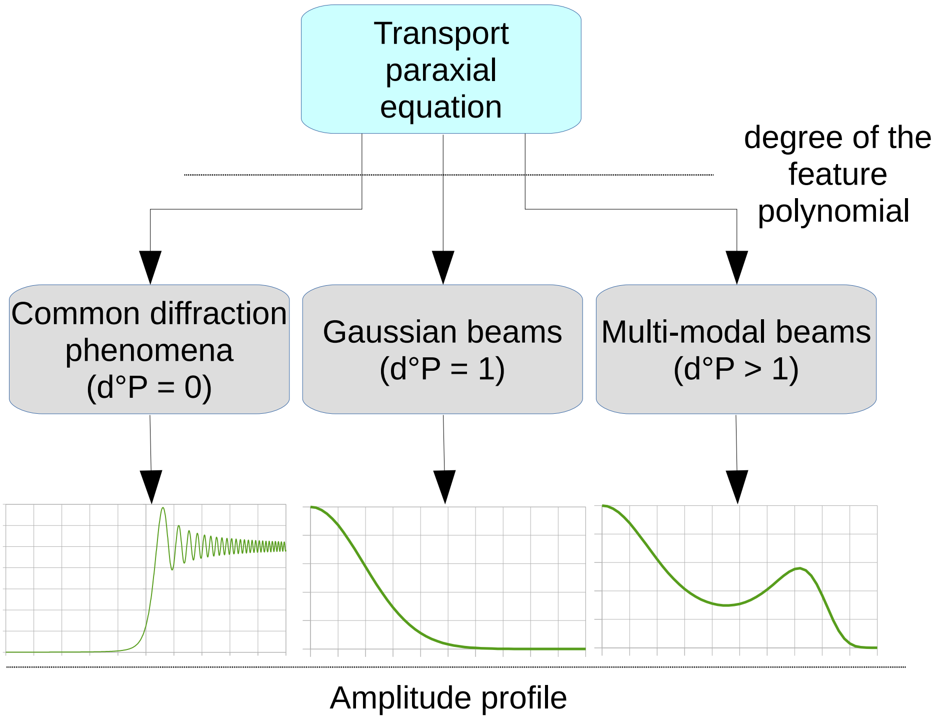

Fig. S2: Simulated possibilities of the transport paraxial equation.

The degree of the feature polynomial drives the beam type: non-Gaussian beams are obtained when the degree equals zero, and astigmatic Gaussian beams with adjustable waists are derived when the degree equals one. Therefore, the convolution formula (9) of the Fresnel diffraction[1] describes the propagation of non-Gaussian beams between two points M and P located at the optical axis.

$$E_P = E_M * E_0 \text{ with } E_0(r, z) = \frac{1}{z} e^{\frac{ikr^2}{2z}} \quad (9)$$

Ray tracing for a perfectly conducting rectangular half plane

Several ray tracing software programs possess additional modules to simulate the light wave propagation from the transport paraxial equation. Modules can be programmed[3] from the Fresnel diffraction formula (9) or from the sum of astigmatic Gaussian beams[4].

In particular, optical devices containing perfectly conducting rectangular half-plane can be shown in 3D images by the “ray tracing” module[5] (Fig. S3).

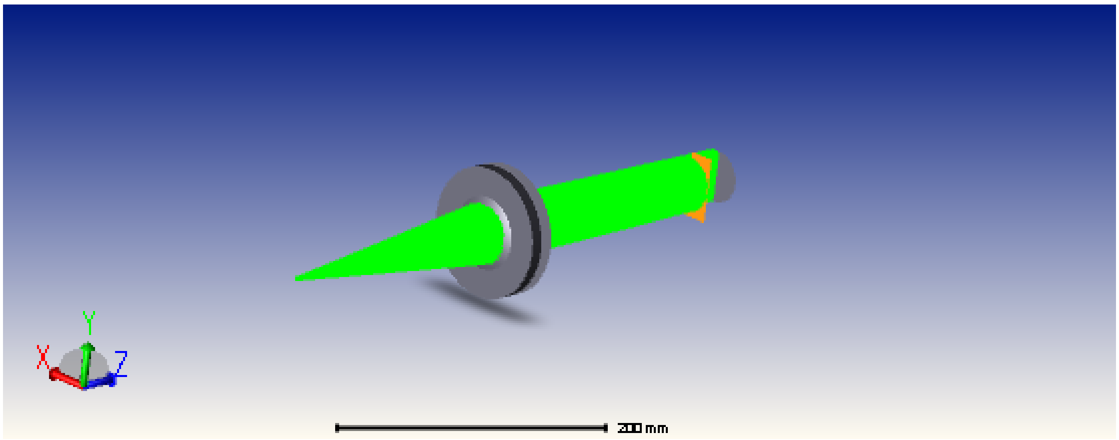

Fig. S3: Perfectly conducting half plane[5].

The simulation[5] of an optical device is shown in an irradiance image by the “propagation” module[5] (Fig. S4).

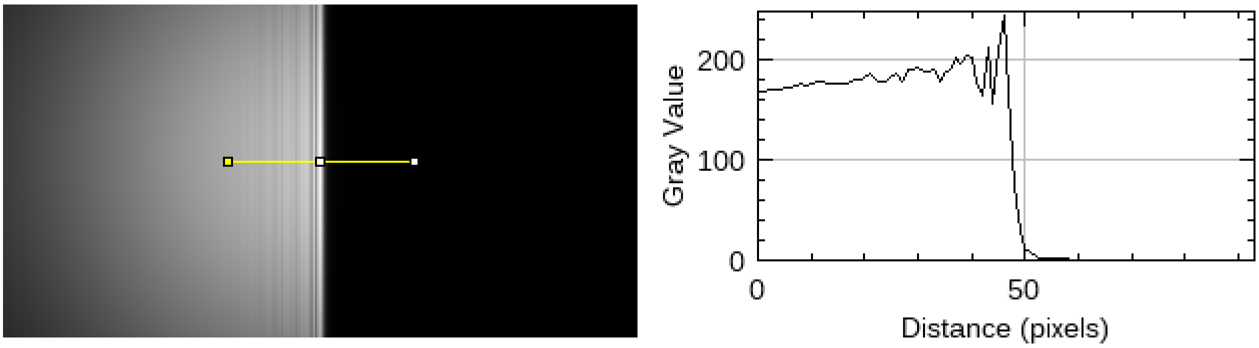

Fig. S4: at left, a piece of the irradiance image[5], and at right, its associated profile.

The profile in the radiation diagram given by the diffraction of a perfectly conducting half-plane (*supplementary note 4, at the right down, Fig. S3*) is similar to an irradiance profile (*at the left, Fig. S4*).

References

1. Born (M.), Wolf (E.), Principes of Optics, Book, 6th edition, *Pergamon Press , Inc* (1980).

2. Zuo (C.), Li (J.), Sun (J.), Fan (Y.), Zhang (J.), Lu (L.), Zhang (R.), Wang (B.), Huang (L.), Chen (Q.), Transport of intensity equation: a tutorial, Optics and Lasers in Engineering, 135:106187 (2020).

3. Aumeyr (T.), Billing (M.G.), Bobb (L.M.), Bolzon (B.), Karataev (P.), Lefevre (T.), Mazzoni (S.), Zemax Simulations of Transition and Diffraction Radiation, J. Phys.: Conf. Ser. 517 012026 (2014).

4. Harvey (JE.), Irvin (RG.), Pfisterer (RN.), Modeling physical optics phenomena by complex ray tracing, Optical Engineering 54(3), 035105 (2015).

5. Ikeda (K.), Gay (S.), OpticStudio TrueFreeform™ optimization for complex illumination systems, Light-Emitting Devices, Materials, and Applications, Proceedings Vol. 11302 (2020).
