# Supplementary material for: A new approach for microstructure imaging
Source: Sci Rep. 2022 Nov 15;12:19565. doi: 10.1038/s41598-022-24176-8 (PMC9666525; doi:10.1038/s41598-022-24176-8)
Supplement: Supplementary file 4 — Supplementary Information 4. [file 41598_2022_24176_MOESM4_ESM.pdf]

Diffraction by a perfectly conducting half-plane

Benoît Plancoulaine[1,2,*], Allan Rasmusson[1,3], Christophe Labbé[4], Richard Levenson[5], Arvydas Laurinavicius[1,3].

1 Institute of Biomedical Sciences, Faculty of Medicine, Vilnius University, Vilnius, Lithuania.

2 ANTICIPE, INSERM, University Caen Normandy, Cancer Center F. Baclesse, Caen, France.

3 National Center of Pathology, Affiliate of Vilnius University Hospital Santaros Clinics, Vilnius, Lithuania.

4 CIMAP, CEA, CNRS, ENSICAEN, University Caen Normandy, Caen, France.

5 Department of Pathology and Laboratory Medicine, UC Davis Health, Sacramento, CA, USA.

Introduction

Ray tracing is an effective tool for studying object illumination and is based on the eikonal nonlinear partial differential and transport equations for addressing optical issues[1]. However, the impact of diffraction is often not considered because the two laws of geometric optics do not describe this aspect directly.

The model describing the diffraction of a perfectly conducting half-plane was initially studied by Gouy[2] in 1883 and then by Poincaré[3] in 1892. Sommerfeld's elegant computational approach[4, 5], based on Helmholtz's equation dating from 1895, provided a solution applying to a homogeneous medium for which initial boundary conditions are known.

However, a solution exists for the Sommerfeld diffraction problem in the case of a perfectly conducting half-plane using the eikonal and transport equations in cylindrical parabolic coordinates.

Light waves in the neighborhood of a half-plane

Lighting a perfectly conducting half-plane with a planar wave results in an electric field that is rectilinearly polarized along the z axis (Fig. S1).

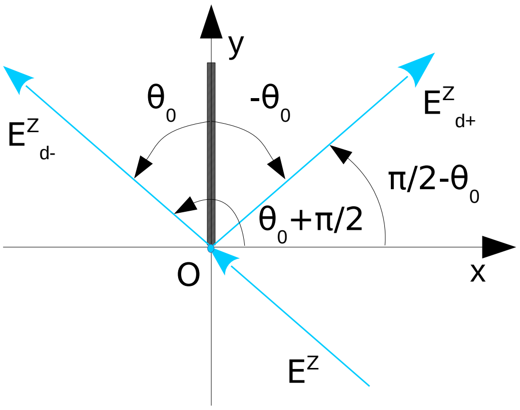

Fig. S1: Lighting of a perfectly conducting half-plane.

When the half-plane is placed along the positive y axis, the incident wave is transmitted along $E_-^z = E_{0-}^z e^{i\phi_-}$ making the angle θ_0 with the y axis, and the

wave is reflected along $E^z_+ = E^z_{0+} e^{i\phi_+}$, generating a wave at the opposite angle $-\theta_0$ with the y axis, according to the Snell-Descartes law.

The phase of the transmitted wave is given by (1).

$$\phi_- = k\left(x\cos\left(\theta_0 + \frac{\pi}{2}\right) + y\sin\left(\theta_0 + \frac{\pi}{2}\right)\right) = -k\left(x\sin(\theta_0) - y\cos(\theta_0)\right) \quad (1)$$

and the phase of the reflected wave is given by (2).

$$\phi_+ = k\left(x\cos\left(\frac{\pi}{2} - \theta_0\right) + y\sin\left(\frac{\pi}{2} - \theta_0\right)\right) = k\left(x\sin(\theta_0) + y\cos(\theta_0)\right) \quad (2)$$

where k is the modulus of the wave vector.

Cylindrical parabolic coordinate system

Cylindrical parabolic coordinates[5] (ξ, η, z) are defined as (3).

$$\left(x = \xi\eta,\ y = \frac{-\xi^2 + \eta^2}{2},\ z\right) \quad (3)$$

where (x, y, z) are the Cartesian coordinates (Fig. S2).

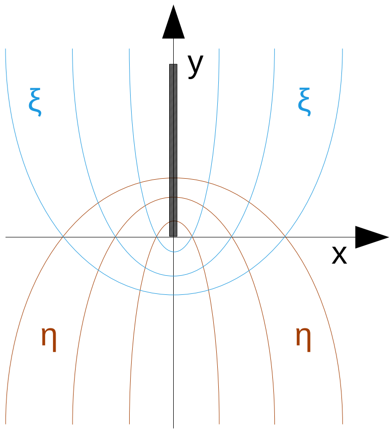

Fig. S2: Parabolic coordinates.

Moreover, the relationships (4) allow conversion to cylindrical coordinates (r, θ, z).

$$\xi = \sqrt{2r}\sin\left(\frac{\pi}{4} - \frac{\theta}{2}\right) \text{ and } \eta = \sqrt{2r}\cos\left(\frac{\pi}{4} - \frac{\theta}{2}\right) \quad (4)$$

The differential operators, namely, gradient, divergence and Laplacian, are respectively defined for parabolic coordinates by (5, 6, 7).

$$\vec{\nabla}(\phi) = \left[\frac{1}{\sqrt{\xi^2+\eta^2}}\frac{\partial\phi}{\partial\xi}, \frac{1}{\sqrt{\xi^2+\eta^2}}\frac{\partial\phi}{\partial\eta}, \frac{\partial\phi}{\partial z}\right] \quad (5)$$

$$\vec{\nabla}\cdot\vec{A} = \frac{1}{\xi^2+\eta^2}\left[\frac{\partial}{\partial\xi}\left(\sqrt{\xi^2+\eta^2}A_\xi\right) + \frac{\partial}{\partial\eta}\left(\sqrt{\xi^2+\eta^2}A_\eta\right)\right] + \frac{\partial A_z}{\partial z} \quad (6)$$

$$\vec{\nabla}\cdot\vec{\nabla}(\phi) = \Delta(\phi) = \frac{1}{\xi^2+\eta^2}\left[\frac{\partial^2\phi}{\partial\xi^2} + \frac{\partial^2\phi}{\partial\eta^2}\right] + \frac{\partial^2\phi}{\partial z^2} \quad (7)$$

Using these coordinates, the phase expressions become (8) for the transmitted electric field and (9) for the reflected electric field.

$$\phi_{-}=-k\left(\xi\eta\sin(\theta_0)-\frac{1}{2}(-\xi^2+\eta^2)\cos(\theta_0)\right) \quad (8)$$

$$\phi_{+}=k\left(\xi\eta\sin(\theta_0)+\frac{1}{2}(-\xi^2+\eta^2)\cos(\theta_0)\right) \quad (9)$$

Computations without straight-edge effect

First, the domain of geometric optics is considered and the computations are performed without incorporation of any straight-edge effect.

The two partial derivatives of the transmitted electric field phase are given by (10).

$$\frac{\partial\phi_{-}}{\partial\xi}=-k(\eta\sin(\theta_0)+\xi\cos(\theta_0)) \text{ and } \frac{\partial\phi_{-}}{\partial\eta}=-k(\xi\sin(\theta_0)-\eta\cos(\theta_0)) \quad (10)$$

The addition of these partial derivatives yields $\frac{1}{\xi^2+\eta^2}\left[\left(\frac{\partial\phi_{-}}{\partial\xi}\right)^2+\left(\frac{\partial\phi_{-}}{\partial\eta}\right)^2\right]=k^2$ which fulfills the eikonal equation (*supplementary note 1*). Similarly, the partial derivatives of the reflected electric field phase are given by (11).

$$\frac{\partial\phi_{+}}{\partial\xi}=k(\eta\sin(\theta_0)-\xi\cos(\theta_0)) \text{ and } \frac{\partial\phi_{+}}{\partial\eta}=k(\xi\sin(\theta_0)+\eta\cos(\theta_0)) \quad (11)$$

and in similar fashion, the summation of these gives $\frac{1}{\xi^2+\eta^2}\left[\left(\frac{\partial\phi_{+}}{\partial\xi}\right)^2+\left(\frac{\partial\phi_{+}}{\partial\eta}\right)^2\right]=k^2$ which also fulfills the eikonal equation.

The amplitude is found from the transport (*supplementary note 1*) by considering the transmitted and reflective parts separately. Regarding the amplitude E_{0-}^{z} for the transmitted electric field, the two parts (12)

$$\begin{cases}\Delta(\phi_{-})=0\\ \vec{\nabla}(\phi_{-})\cdot\vec{\nabla}(E_{0-}^{z})=-\frac{k}{\xi^2+\eta^2}\left[(\eta\sin(\theta_0)+\xi\cos(\theta_0))\frac{\partial E_{0-}^{z}}{\partial\xi}+(\xi\sin(\theta_0)-\eta\cos(\theta_0))\frac{\partial E_{0-}^{z}}{\partial\eta}\right]\end{cases} \quad (12)$$

are substituted into the transport equation, leading to (13) using the change of variable $\zeta_{d-}=\sqrt{k}\left[\eta\cos\left(\frac{\theta_0}{2}\right)-\xi\sin\left(\frac{\theta_0}{2}\right)\right]$.

$$\zeta_{d-}\frac{\partial E_{0-}^{z}}{\partial\zeta_{d-}}=0 \quad (13)$$

After integration of the previous equation (13) the amplitude is seen to be constant $E_{0-}^{z}=E_{0-}^{z}(0)$ as is typical for a planar wave.

Concerning the amplitude E_{0+}^{z} of the reflected electric field, the two parts (14)

$$\begin{cases} \Delta(\phi_+)=0 \\ \vec{\nabla}(\phi_+)\cdot\vec{\nabla}(E_{0+}^z)=\frac{k}{\xi^2+\eta^2}\left[(\eta\sin(\theta_0)-\xi\cos(\theta_0))\frac{\partial E_{0+}^z}{\partial\xi}+(\xi\sin(\theta_0)+\eta\cos(\theta_0))\frac{\partial E_{0+}^z}{\partial\eta}\right] \end{cases} \quad (14)$$

are similarly substituted into the transport equation, leading to (15) using the change of variable by $\zeta_{d+}=\sqrt{k}\left[\xi\sin\left(\frac{\theta_0}{2}\right)+\eta\cos\left(\frac{\theta_0}{2}\right)\right]$.

$$\zeta_{d+}\frac{\partial E_{0+}^z}{\partial\zeta_{d+}}=0 \quad (15)$$

Again, the amplitude is a constant $E_{0+}^z=E_{0+}^z(0)$ after integration.

These initial results permit the definition of two new variables, ζ_{d-} and ζ_{d+}, which had previously been used by Sommerfeld to adjust the phase of the planar waves.

Computations including straight-edge effect

We now take the effect of the straight edge of the half-plane into account by means of cylindrical parabolic coordinates (Fig. S2), altering the transmitted electric field phase by $\phi_{d-}=\phi_- -\zeta_{d-}^2$ and the reflected electric field phase by $\phi_{d+}=\phi_+ -\zeta_{d+}^2$. The derivations follow the same outline used in the previous section.

The computation of the norm of the reflected phase with the added Sommerfeld term, ζ_{d-}^2, is (16).

$$\|\vec{\nabla}(\phi_{d-})\|^2=\|\vec{\nabla}(\phi_-)\|^2-2\,\vec{\nabla}(\phi_-)\cdot\vec{\nabla}(\zeta_{d-}^2)+\|\vec{\nabla}(\zeta_{d-}^2)\|^2 \quad (16)$$

This must fulfill the eikonal equation, and the involved terms are in (17).

$$\begin{cases} \frac{1}{\xi^2+\eta^2}\left[\left(\frac{\partial\phi_-}{\partial\xi}\right)^2+\left(\frac{\partial\phi_-}{\partial\eta}\right)^2\right]=k^2 \\ \left(\frac{\partial\zeta_{d-}^2}{\partial\xi}\right)^2+\left(\frac{\partial\zeta_{d-}^2}{\partial\eta}\right)^2=2k^2[\xi^2(1-\cos(\theta_0))-2\xi\eta\sin(\theta_0)+\eta^2(1+\cos(\theta_0))] \\ -2\left[\frac{\partial\phi_-}{\partial\xi}\frac{\partial\zeta_{d-}^2}{\partial\xi}+\frac{\partial\phi_-}{\partial\eta}\frac{\partial\zeta_{d-}^2}{\partial\eta}\right]=-\left[\left(\frac{\partial\zeta_{d-}^2}{\partial\xi}\right)^2+\left(\frac{\partial\zeta_{d-}^2}{\partial\eta}\right)^2\right] \end{cases} \quad (17)$$

The sum of these parts gives (18).

$$\left(\frac{\partial\phi_-}{\partial\xi}\right)^2+\left(\frac{\partial\phi_-}{\partial\eta}\right)^2=k^2(\xi^2+\eta^2) \quad (18)$$

Therefore, when all parts are summed, the eikonal equation is fulfilled for the transmitted electric field phase.

The equivalent computation of the norm of the reflected phase with the added Sommerfeld term, ζ_{d+}^2 , is (19).

$$\|\vec{\nabla}(\phi_{d+})\|^2=\|\vec{\nabla}(\phi_+)\|^2-2\,\vec{\nabla}(\phi_+)\cdot\vec{\nabla}(\zeta_{d+}^2)+\|\vec{\nabla}(\zeta_{d-}^2)\|^2 \quad (19)$$

which must still fulfill the eikonal equation, similarly involves (20).

$$\begin{cases} \left(\frac{\partial\phi_+}{\partial\xi}\right)^2+\left(\frac{\partial\phi_+}{\partial\eta}\right)^2=k^2(\xi^2+\eta^2) \\ \left(\frac{\partial\zeta_{d+}^2}{\partial\xi}\right)^2+\left(\frac{\partial\zeta_{d+}^2}{\partial\eta}\right)^2=2k^2[\xi^2(1-\cos(\theta_0))+2\xi\eta\sin(\theta_0)+\eta^2(1+\cos(\theta_0))] \\ -2\left[\frac{\partial\phi_+}{\partial\xi}\frac{\partial\zeta_{d+}^2}{\partial\xi}+\frac{\partial\phi_+}{\partial\eta}\frac{\partial\zeta_{d+}^2}{\partial\eta}\right]=-\left[\left(\frac{\partial\zeta_{d+}^2}{\partial\xi}\right)^2+\left(\frac{\partial\zeta_{d+}^2}{\partial\eta}\right)^2\right] \end{cases} \quad (20)$$

The sum of these parts gives (21).

$$\left(\frac{\partial\phi_+}{\partial\xi}\right)^2+\left(\frac{\partial\phi_+}{\partial\eta}\right)^2=k^2(\xi^2+\eta^2) \quad (21)$$

which shows that the eikonal equation is met again for the reflected electric field phase. In conclusion, the transmitted and the reflected electric fields fulfills the eikonal equation using the different Sommerfeld's adjustments ζ_{d-}^2 and ζ_{d+}^2 .

The amplitude can again be determined using the transport equation. The derivation of the transmitted electric field amplitude E_{0d-}^z involves (22).

$$\begin{cases} \Delta(\phi_-)-\Delta(\zeta_{d-}^2)=-\frac{2.k}{\xi^2+\eta^2} \\ \vec{\nabla}(\phi_- -\zeta_{d-}^2)\cdot\vec{\nabla}(E_{0d-}^z)=-\frac{k}{\xi^2+\eta^2}\left[\xi\frac{\partial E_{0d-}^z}{\partial\xi}+\eta\frac{\partial E_{0d-}^z}{\partial\eta}\right] \end{cases} \quad (22)$$

These are then inserted into the transport equation to give (23) using the change of variable $\zeta_{d-}=\sqrt{k}\left[\eta\cos\left(\frac{\theta_0}{2}\right)-\xi\sin\left(\frac{\theta_0}{2}\right)\right]$.

$$E_{0d-}^z+\zeta_{d-}\frac{\partial E_{0d-}^z}{\partial\zeta_{d-}}=0 \quad (23)$$

Finally, the transmitted electric field amplitude becomes $E_{0d-}^z=\frac{E_{0d-}^z(0)}{\zeta_{d-}}$ with $E_{0d-}^z(0)$ being a constant after the integration of the previous equation (23).

Concerning the reflected electric field, the computation of the amplitude E_{0d+}^z involves (24).

$$\begin{cases} \Delta(\phi_+)-\Delta(\zeta_{d+}^2)=-\dfrac{2.k}{\xi^2+\eta^2} \\ \vec{\nabla}(\phi_+-\zeta_{d+}^2)\cdot\vec{\nabla}(E_{0d+}^z)=-\dfrac{k}{\xi^2+\eta^2}\left[\xi\dfrac{\partial E_{0d+}^z}{\partial\xi}+\eta\dfrac{\partial E_{0d+}^z}{\partial\eta}\right] \end{cases} \quad (24)$$

The results are put into the transport equation to yield (25) using the change of variable $\zeta_{d+}=\sqrt{k}\left[\xi\sin\left(\frac{\theta_0}{2}\right)+\eta\cos\left(\frac{\theta_0}{2}\right)\right]$.

$$E_{0d+}^z+\zeta_{d+}\frac{\partial E_{0d+}^z}{\partial\zeta_{d+}}=0 \quad (25)$$

Finally, the transmitted electric field amplitude becomes $E_{0d+}^z=\frac{E_{0d+}^z(0)}{\zeta_{d+}}$, with $E_{0d+}^z(0)$ a constant, after the integration of the previous equation (25).

Electric field diffracted by the edge of a half-plane

The diffracted field is computed using cylindrical coordinates. The transmitted diffracted electric field $E_{d-}^z=E_{0d-}^z(0)e^{i\phi_-}\frac{e^{-i\zeta_{d-}^2}}{\zeta_{d-}}$ becomes (26).

$$E_{d-}^z=\frac{E_{0d-}^z(0)}{\sqrt{2kr}}\frac{e^{-ikr}}{\cos((\theta-\theta_0)/2-\pi/4)} \quad (26)$$

because of the relationships $\zeta_{d-}=\sqrt{2kr}\cos\left(\frac{\theta-\theta_0}{2}-\frac{\pi}{4}\right)$, $\phi_{d-}=\phi_--\zeta_{d-}^2=-kr$ and $\phi_-=kr\sin(\theta-\theta_0)$.

Similarly, the reflected diffracted electric field $E_{d+}^z=E_{0d+}^z(0)e^{i\phi_+}\frac{e^{-i\zeta_{d+}^2}}{\zeta_{d+}}$ becomes (27).

$$E_{d+}^z=\frac{E_{0d+}^z(0)}{\sqrt{2kr}}\frac{e^{-ikr}}{\cos((\theta+\theta_0)/2-\pi/4)} \quad (27)$$

because of the relationships $\zeta_{d+}=\sqrt{2kr}\cos\left(\frac{\theta+\theta_0}{2}-\frac{\pi}{4}\right)$, $\phi_{d+}=\phi_+-\zeta_{d+}^2=-kr$ and $\phi_+=kr\sin(\theta+\theta_0)$.

The total diffracted electric field is the sum of the transmitted and reflected diffracted electric field and it is given by (28).

$$E_d^z=\frac{1}{\sqrt{2k}}\frac{e^{-ikr}}{\sqrt{r}}\left[\frac{E_{0d+}^z(0)}{\cos((\theta+\theta_0)/2-\pi/4)}+\frac{E_{0d-}^z(0)}{\cos((\theta-\theta_0)/2-\pi/4)}\right] \quad (28)$$

The boundary conditions are chosen so that $E_d^z=0$ along the y axis. When the angle $\theta=\pi/2$ is taken, the relationship between the constants $E_{0d-}^z(0)$ and

$E^z_{0d+}(0)$ is given by $E^z_{0d+}(0) = -E^z_{0d-}(0)$ and when the angle $\theta = -\pi/2$, the relationship between the constants $E^z_{0d-}(0)$ and $E^z_{0d+}(0)$ is given by $E^z_{0d+}(0) = E^z_{0d-}(0)$. Finally, the total diffracted electric field can be expressed as (29).

$$E^z_d = \frac{E^z_{0d}(0)}{\sqrt{2k}} \frac{e^{-ikr}}{\sqrt{r}} \left[\frac{1}{\cos((\theta+\theta_0)/2 - \pi/4)} \pm \frac{1}{\cos((\theta-\theta_0)/2 - \pi/4)} \right] \quad (29)$$

The formula of this cylindrical wave is the same as the diffraction formula for the edge of the half-plane except for a constant due to differing boundary conditions[6].

Radiation diagram

Radiation diagrams depict irradiance by directions for a given coordinate r. The electric field must consider all fields present for these diagrams (Fig S3). Several electric fields therefore interfere: the incident field, the refracted filed in the sense of geometric optics and the total diffracted electric field.

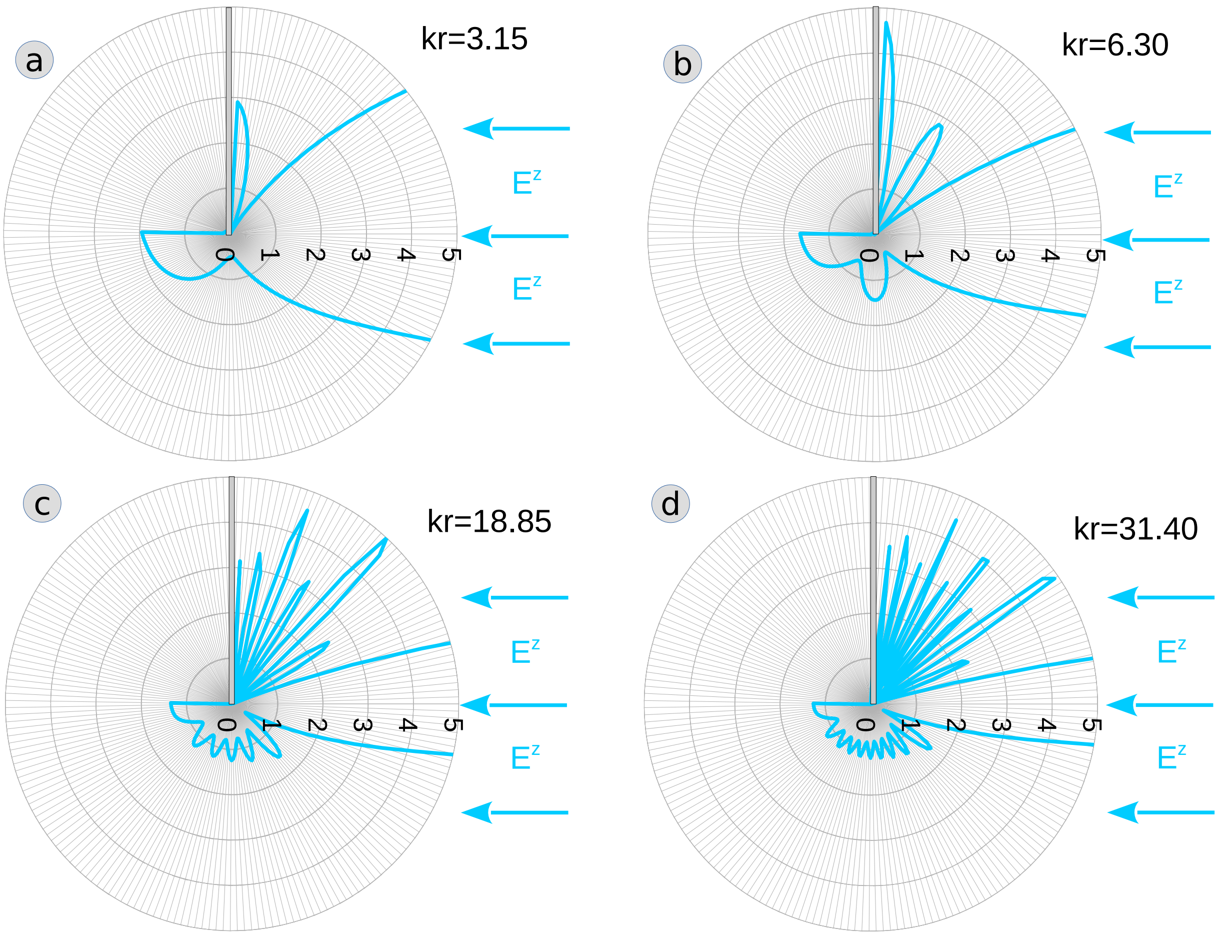

Fig. S3: Four radiation diagrams: a,b given for near fields and c,d given for far fields.

References

1. Runborg (O.), Review Article: Mathematical Models and Numerical Methods for High Frequency Waves, Commun. Comput. Phys., 2:827-880 (2007).

2. Gouy (M.), Sur la polarisation de la lumière diffractée, Compt. Rend. Acad. Sci. 96, 697 (1883, French language).

3. Poincaré (H.), Sur la Polarisation par Diffraction, Acta Math. 16, 297 (1892, French language).

4. Sommerfeld (A.), Mathematische Theorie der Diffraction, Math. Ann. 47, 317 (German language, 1896).

5. McDonald (KT.), Sommerfeld's Diffraction Problem, Princeton University, Princeton, (2012).

6. Keller (JB.), Diffraction by an Aperture, Journal of applied physics, 28(4):426, 444 (1957).
