# Supplementary material for: A new approach for microstructure imaging
Source: Sci Rep. 2022 Nov 15;12:19565. doi: 10.1038/s41598-022-24176-8 (PMC9666525; doi:10.1038/s41598-022-24176-8)
Supplement: Supplementary file 5 — Supplementary Information 5. [file 41598_2022_24176_MOESM5_ESM.pdf]

Real irradiance computed by means of the phase

Benoît Plancoulaine[1,2,*], Allan Rasmusson[1,3], Christophe Labbé[4], Richard Levenson[5], Arvydas Laurinavicius[1,3].

1 Institute of Biomedical Sciences, Faculty of Medicine, Vilnius University, Vilnius, Lithuania.

2 ANTICIPE, INSERM, University Caen Normandy, Cancer Center F. Baclesse, Caen, France.

3 National Center of Pathology, Affiliate of Vilnius University Hospital Santaros Clinics, Vilnius, Lithuania.

4 CIMAP, CEA, CNRS, ENSICAEN, University Caen Normandy, Caen, France.

5 Department of Pathology and Laboratory Medicine, UC Davis Health, Sacramento, CA, USA.

Introduction

Solving the amplitude simultaneously with the phase is a consistent issue in physics. For example, the same mathematical computations can be applied to solve the Schrödinger or the Helzmoltz equation[1]. In this way, the eikonal and amplitude equations (*supplementary note 1*) can estimate bright effects in microstructures.

Rewording equations for scalar electric field

The eikonal and transport equation are rewritten to the differential system (1) where ϕ is $\phi = k_0 \delta_0$ with k_0 the modulus of the wave vector in the vacuum and δ_0 the eikonal; n is the refractive index, and E_0 is the amplitude of the scalar electric field.

$$\begin{cases} |\vec{\nabla}(\delta_0)| = n \\ \vec{\nabla} \cdot (E_0^2 \vec{\nabla}(\delta_0)) = 0 \end{cases} \quad (1)$$

The solution is given in Cartesian coordinates (x, y, z) whose z acts as the propagation time. Therefore, the first equation (1) successively becomes the resolving equation (2).

$$\left(\frac{\partial \delta_0}{\partial x}\right)^2 + \left(\frac{\partial \delta_0}{\partial y}\right)^2 + \left(\frac{\partial \delta_0}{\partial z}\right)^2 = n^2 \text{ then } \frac{\partial \delta_0}{\partial z} - \sqrt{n^2 - \left(\frac{\partial \delta_0}{\partial x}\right)^2 - \left(\frac{\partial \delta_0}{\partial y}\right)^2} = 0 \quad (2)$$

The second equation (1) evolves to times: the gradient part (3)

$$E_0^2 \vec{\nabla}_x(\delta_0) = \left[E_0^2 \frac{\partial \delta_0}{\partial x}, E_0^2 \frac{\partial \delta_0}{\partial y}, E_0^2 \frac{\partial \delta_0}{\partial z}\right] = [a\mu, b\mu, \mu] \quad (3)$$

where a is given by $a = \left(\frac{\partial \delta_0}{\partial x}\right)\left(\frac{\partial \delta_0}{\partial z}\right)^{-1}$, b is given by $b = \left(\frac{\partial \delta_0}{\partial y}\right)\left(\frac{\partial \delta_0}{\partial z}\right)^{-1}$ and μ is given by $\mu = E_0^2 \frac{\partial \delta_0}{\partial z}$ and then the divergent part (4).

$$\frac{\partial a\mu}{\partial x}+\frac{\partial b\mu}{\partial y}+\frac{\partial \mu}{\partial z}=0 \quad (4)$$

It should be noted that a, b and $I_0=E_0^2$ depend on the eikonal δ_0, and a unique solution exists for this differential system of the two equations (2) and (4) for very general initial boundaries[1].

Numerical schema of Lax-Friedrichs type

The differential system obtained from the two equations (2) and (4) can be computed with equations of differences. Regular sampled grids are drawn in a three-dimensional space: the x and y coordinates are sampled with the same step Δxy giving sampled plans $\mathbb{R}^2=\bigcup_{i\in\mathbb{Z}}[x_i, x_{i+1}]\times\bigcup_{j\in\mathbb{Z}}[y_j, y_{j+1}]$, and the z coordinates are also sampled with the step Δz giving the sampled axis $\mathbb{R}^+=\bigcup_{k\in\mathbb{N}}[z^k, z^{k+1}]$. Therefore, each point in three-dimensional space is shown by $(x_i, y_j, z^k)=(i\Delta xy, j\Delta xy, k\Delta z)$ and belongs to a three-dimensional matrix.

Numerical schema for the eikonal equation (2) resolves from the equation of the differences (5).

$$\frac{u_{i,j}^{k+1}-u_{i,j}^k}{\Delta z}-\sqrt{n^2(x_i, y_j, z^k)-\left(\frac{u_{i+1,j}^k-u_{i,j}^k}{\Delta xy}\right)^2-\left(\frac{u_{i,j+1}^k-u_{i,j}^k}{\Delta xy}\right)^2}=0 \quad (5)$$

where the sample $u_{i,j}^k$ is $u_{i,j}^k=\delta_0(x_i, y_j, z^k)$ which is modified to the new relationship (6)

$$u_{i,j}^{k+1}=u_{i,j}^k+\frac{\Delta z}{\Delta xy}\sqrt{(n(x_i, y_j, z^k)\Delta xy)^2-(u_{i+1,j}^k-u_{i,j}^k)^2-(u_{i,j+1}^k-u_{i,j}^k)^2} \quad (6)$$

The numerical Lax-Friedrichs[2] type schema substitutes each sample by centering with its nearest neighbors (7).

$$u_{i+1,j}^k=\frac{u_{i+1,j+1}^k+u_{i+1,j-1}^k}{2}, \quad u_{i,j+1}^k=\frac{u_{i+1,j+1}^k+u_{i-1,j+1}^k}{2}, \quad u_{i,j}^k=\frac{u_{i+1,j+1}^k+u_{i-1,j+1}^k+u_{i+1,j-1}^k+u_{i-1,j-1}^k}{4}$$

(7)

It should be noted the ratio $\frac{\Delta z}{\Delta xy}$ keeps a fixed value even if the differences becomes very small.

Numerical schema for the transport equation (4) resolve from the equation of the differences (8).

$$v_j^{k+1}=v_{i,j}^k-\frac{\Delta z}{\Delta xy}[(f(v_{i,j+1}^k)-f(v_{i,j}^k))+(g(v_{i+1,j}^k)-g(v_{i,j}^k))] \quad (8)$$

where the sample $v_{i,j}^k$ is $v_{i,j}^k=\mu(x_i, y_j, z^k)$ and the functions $f(a\mu)$ and $g(b\mu)$ are $f(a\mu)=a\mu$ and $g(b\mu)=b\mu$, respectively. The numerical Lax-Friedrichs type schema allow for computing the relationship (8). Herein, these computations

require coupling for each propagation time z^k with the computation of the eikonal equation. The coefficients a and b and the irradiance I_0 use the partial differential equations $\frac{\partial \delta_0}{\partial x}$, $\frac{\partial \delta_0}{\partial y}$ and $\frac{\partial \delta_0}{\partial z}$ which are associated respectively to $\frac{u_{i+1,j}^k - u_{i,j}^k}{\Delta xy}$, $\frac{u_{i,j+1}^k - u_{i,j}^k}{\Delta xy}$ and $\frac{u_{i,j}^{k+1} - u_{i,j}^k}{\Delta z}$ with Δz deduced from the ratio $\frac{\Delta z}{\Delta xy}$. The numerical Lax-Friedrichs[2] type schema are renewed for these computations.

The numerical Lax-Friedrichs type schema are used in order to warrant better numerical convergence. This approach is feasible and efficient in several practical situations[1].

Smooth wedge simulations

Smooth wedges are used to illustrate results of the numerical schema (Fig. S1).

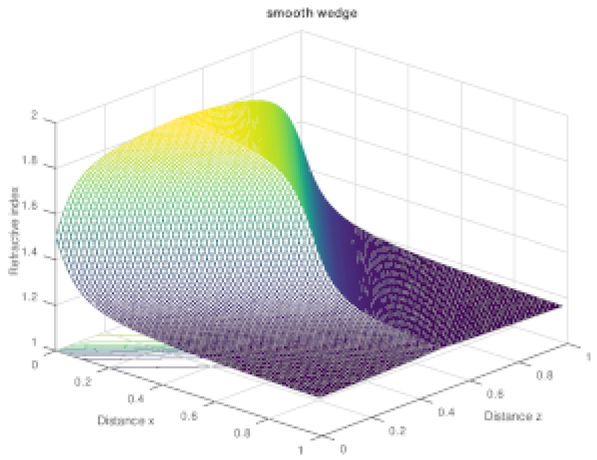

Fig. S1: Reflective index of a smooth wedge in a 2D plane.

The 2D numerical schema computes the phase and the irradiance (Fig. S2).

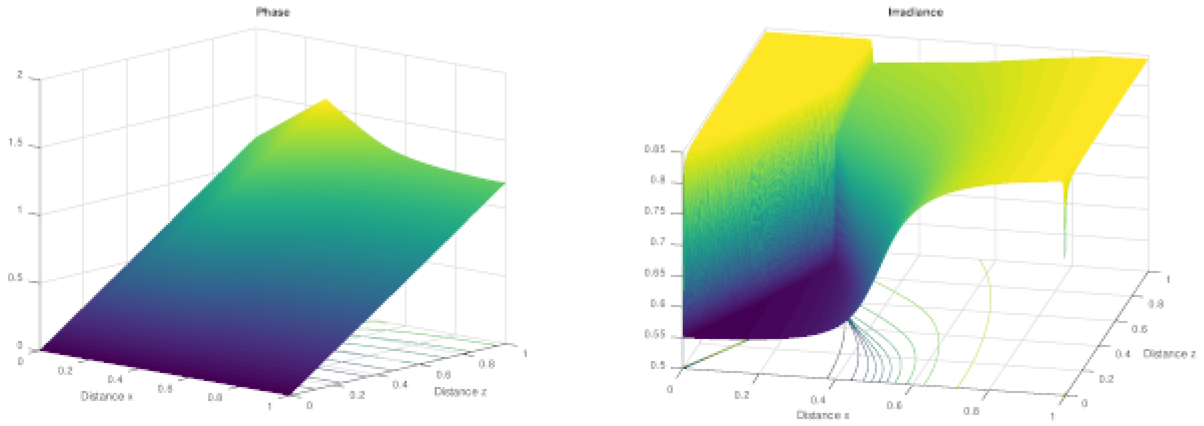

Fig. S2: at the left, the phase image and at the right, the irradiance image.

The 3D numerical schema allows to build images in a *xy* plane to study the irradiance (Fig. S3).

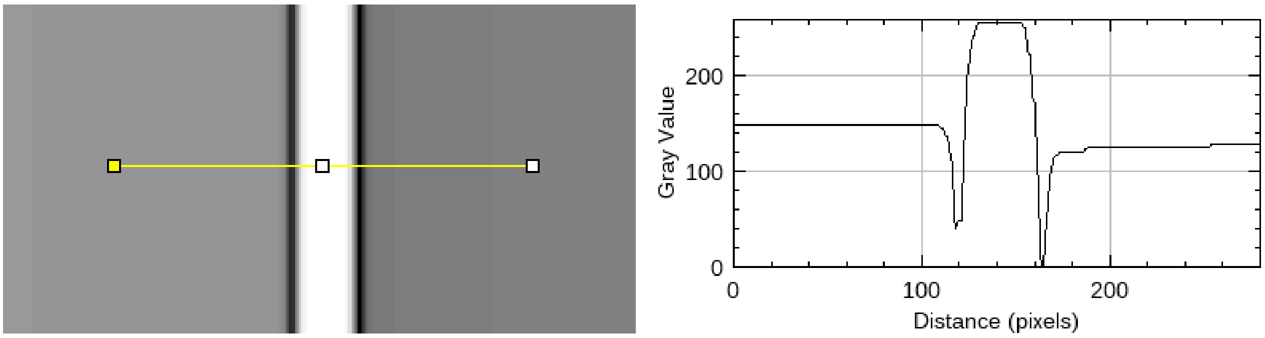

Fig. S3: at the left, a piece of the irradiance image and at the right, its associated profile.

The profile in the radiation diagram given by the diffraction of a perfectly conducting half-plane (*supplementary note 4, at the right top, Fig. S3*) is similar to the irradiance profile (*at the left, Fig. S3*).

References

1. Gosse (L.), James (F.), Convergence results for an inhomogeneous system arising in various high frequency approximations, Numer. Math. 90: 721–753 (2002).

2. Breuss (M.), The correct use of the Lax-Friedrichs method, ESAIM: M2AN, 38(3):519-540 (2004).
